# Guidelines of the Italian Society for Virology on HPV testing and vaccination for cervical cancer prevention

**DOI:** 10.1186/1750-9378-3-14

**Published:** 2008-12-16

**Authors:** Luisa Barzon, Colomba Giorgi, Franco M Buonaguro, Giorgio Palù, the Italian Society for Virology

**Affiliations:** 1 Department of Histology, Microbiology, and Medical Biotechnologies, University of Padova, 35121 Padova, Italy; 2 Department of Infectious, Parasitic, and Immunomediated Diseases, Istituto Superiore di Sanità, Roma, Italy; 3 Viral Oncology and AIDS Reference Centre, National Cancer Institute, "Fondazione Pascale", Cappella Cangiani, 80131 Napoli, Italy

## Abstract

**Objective:**

To provide guidelines for health-care providers on strategies for cervical cancer prevention based on HPV testing and anti-HPV vaccination.

**Outcomes:**

Overall efficacy of different preventive strategies, assessing reduction in the incidence of invasive cervical cancer and precancerous lesions.

**Evidence:**

Medline and the Cochrane Database were searched for articles in English on subjects related to HPVs, HPV diagnosis, HPV anogenital lesions, cervical cancer, HPV testing, and HPV vaccines, in order to elaborate an up-dated document. Relevant Italian Government publications and position papers from appropriate health and family planning organizations were also reviewed.

**Values:**

The quality of the evidence and ranking of recommendations for practice were rated using criteria defined by SIV, which were adapted from the Canadian Task Force on Preventive Health Care.

## Introduction

This report summarizes recommendations on cervical cancer screening and prevention strategies proposed by experts of the Italian Society for Virology (SIV, Società Italiana di Virologia) on the basis of systematic review of evidence from the scientific literature and current guidelines produced by International Societies, in view of an European consensus strategy for cervical cancer screening, human papillomavirus (HPV) testing, and anti-HPV vaccination programs. The guidelines were discussed with experts in the field (see acknowledgment section) and with SIV members, and reviewed and approved by SIV Scientific Committee, during the annual SIV Conference of 2008, following a specific Round table on the subject. These recommendations recognize the diagnostic value of HPV testing and genotyping in the management of women with abnormal PAP test and in screening programs for prevention of cervical carcinoma and recommend HPV vaccination for primary prevention of cervical cancer. The searching strategies, the study selection criteria and other methodologies adopted for the literature review can be asked to the correspondig author.

## Human papillomavirus and cervical cancer

The demonstration that human papillomavirus (HPV) infection has a pathogenetic role on cervical cancer development and the understanding of the epidemiology of HPV infection and its relationship with the natural history of precancerous lesions and cervical cancer have been crucial in the development of diagnostic and vaccination strategies for prevention of one of the most common and lethal cancers, especially in developing countries.

### Epidemiology of cervical cancer and HPV infection

Epidemiological data on HPV infection and associated disease have been reviewed in a recently published IARC Monograph [1]. Cervical cancer is the second most common and lethal cancer in women worldwide, with estimated 493,000 new cases and 274,000 deaths in 2002 [2]. More than 80% of cervical cancers and related deaths occur in developing countries, where cervical cancer is the most common cancer, accounting for 15% of female cancers, while in developed countries it accounts for only 3.6% of new cancers [2]. Cervical cancer has a relatively low incidence in Europe (less than 15 per 100,000), whereas its incidence is generally over 30 per 100,000 in developing countries [3,4]. The incidence of invasive cervical cancer has a peak at about 20–25 years after the peak age for HPV infection prevalence, and the incidence of cervical intraepithelial neoplasia (CIN) peaks in between [1]. According to the AIRTum (Associazione Italiana dei Registri Tumori) database, in Italy, during 1998–2002, cervical cancer represented 1.6% of all newly diagnosed cancers among females, with an average incidence of 9.8 cases per 100,000 females per year, and represented 0.6% of all cancer deaths among females. It has been estimated that every year 3,418 new cervical carcinomas are diagnosed in Italy; as regards mortality, in 2002 there were 370 deaths due to cervical cancer, and 1,756 deaths due to cancer of the uterus not otherwise specified [5]. Survival rates in patients with cervical cancer vary in different countries, ranging from 73% at 5 years in US [6] and 63% in Europe [7] to 30.5% in sub-Saharan Africa [8]. In Italy, at 5 years from diagnosis of cervical cancer, survival rates range between 78% in Ferrara and 40% in Naples, with an average national survival rate of 65% [5].

Epidemiological and experimental evidence demonstrate that persistent infection with a carcinogenic HPV genotype is a necessary cause of cervical cancer and its precancerous lesions, as suggested by zur Hausen in '70 [9]. Over 120 different HPV genotypes have been identified so far and at least 40 of these HPV types infect the anogenital mucosa [10]. Genital HPVs are designated as *high-risk *or *low-risk *types, based on their association with cervical cancer [11-14]. It should be recognized that the distinction between high-risk and low-risk HPV genotypes is continuously under revision on the basis of new knowledge in the field; therefore the definition of the oncogenicity of some HPVs could change with time, with obvious implications on the definition of the diagnostic standards for HPV infection, as outlined below.

High-risk HPV DNA is virtually detectable in all cervical cancers [15]. Among high-risk or carcinogenic HPVs (currently, HPV types 16, 18, 31, 33, 35, 39, 45, 51, 52, 56, 58, 59) and probably carcinogenic HPVs (HPV types 26, 53, 66, 68, 73, 82) [4,5], HPV-16 is responsible for 60% of cervical cancer, HPV-18 for 10%, HPV-45 and HPV-31 for 4% each, whereas HPV-33, HPV-52, and HPV-58 altogether account for 2% of cervical cancer [12,16]. In high-grade squamous intraepithelial lesions (HSIL), the most common HPV types identified are similar to those in cervical cancer, except for the under-representation of HPV-18 and HPV-45 [16]. In low-grade squamous intraepithelial lesions (LSIL), high-risk HPV DNA can be detected in 30 to 100% of cases in different studies [17]. Again, HPV-16 is the most common type, being detected in 26% of cases, followed by HPV-31 (12%), HPV-51 (11%), HPV-53 and HPV-56 (10%), and many other HPV types [17].

A recent meta-analysis investigated the worldwide prevalence and genotype distribution of HPV DNA in cervical samples from women with normal cytology [18]. This meta-analysis estimated the overall HPV prevalence in women with normal cytology was 10.4% (95% confidence interval [CI], 10.2–10.7%). The prevalence was relatively high in Africa, Central America and Mexico (about 20 to 30%) and lower in northern America, Europe, and Asia (about 8 to 11%). In all world regions, HPV prevalence was highest in women younger than 35 years of age, decreasing in women of older age [18]. In Africa, the Americas, and Europe, a second peak of HPV prevalence was observed in women of older age [18]. Overall, the most common HPV types were HPV-16, HPV-18, HPV-31, HPV-58, and HPV-52, while the most common types in southern Europe were HPV-16, HPV-66, HPV-45, HPV-31, and HPV-42 [18].

In the Italian population, the prevalence of high-risk HPV in women aged 25–70 years has been reported to be about 9%, and HPV-16 was the most common genotype, being detected in 30–60% of HPV-positive women, followed by HPV-66, HPV-45, HPV-31, and HPV-53 [19,20]. HPV genotyping by restriction fragment length polymorphism analysis and direct sequencing showed that, in HSIL, HPV-16 and HPV-31 were the most commonly detected types, followed by HPV-67, HP-87, HPV-61, and HPV-58, while HPV-16, HPV-31, HPV-66, HPV-53, HPV-52, and HPV-56 were the most frequent types in LSIL [21]. By contrast, low-risk HPV-6 and HPV-11 were responsible for about 90% of genital warts [22], whereas the other low-risk HPV types were less frequently associated with anogenital lesions. Data at recruitment from the New Technologies for Cervical Cancer Screening (NTCC) randomized trial, which is ongoing in Italy and includes women attending routine cervical screening programs, showed the rates of high-risk HPV positivity were 13.1% for women aged 25–34 and 5.8% for women aged 35–60, without a second peak of HPV prevalence [23]. In younger women aged 18 to 24, the prevalence of high-risk HPV DNA detection is 17.4% and HPV-16 was the most frequent type, followed by HPV-53, HPV-84, HPV-42, HPV-62, HPV-66, and HPV-89 [24]. A higher prevalence has been reported in a Danish population-based study [25], which showed that about 40% and 45% of women aged 15–19 and 20–24 years, respectively, were infected with high-risk HPV types. HPV-16 was the most common HPV type, followed by HPV-31, HPV-52, and HPV-51 [25]. Like in the Italian population, the prevalence of infection markedly decreased with age, without significant second prevalence peak [25]. The EDITH (Etude de la Distribution des Types d'HPV en France) study investigated the prevalence and distribution of HPV types in France. The results of this study showed that HPV DNA could be detected in 98% of LSIL smears, in 98% of high-grade cervical intraepithelial neoplasia (CIN2/3), and in 97% of invasive cancers [26-28]. In LSIL, the most prevalent genotypes were HPV-66, HPV-16, HPV-53, HPV-51 and HPV-52 [26]; in CIN2/3, HPV-16 was by far the most prevalent genotype (62%), followed by HPV-31, HPV-33, HPV-52, HPV-51, and HPV-58 [27], while the most prevalent genotypes in invasive cervical cancer were HPV-16 (73%) and HPV-18 (19%), followed by HPV-31 (7%), HPV-33, HPV-68, HPV-45, HPV-52, and HPV-58 (4.1-2.3%) [28]. While HPV-16 was the most prevalent type in both squamous cell carcinoma (74%) and adenocarcinoma (64%), HPV-18 was more prevalent in adenocarcinoma (37%) compared to squamous cell carcinoma (16%) [28].

Besides cervical cancer, HPV infection is associated with over 70% of cases of anal carcinoma and squamous oropharyngeal carcinoma, with 50% of vulvar, vaginal, and penile cancer, and, in immunocompromised patients, with about 90% of cutaneous squamous cell cancer [12,29,30]. In Italy, high-risk HPV DNA, mainly HPV-16, was detected in 46% of cases of penile carcinoma [31].

### Pathogenesis

Several basic experimental studies have clarified the mechanisms of HPV oncogenesis [29,32]. In its life cycle, HPV requires infection of epidermal or mucosal epithelial cells of the basal layer, that are still able to proliferate, most probably through wounds or abrasions. In basal layer cells, viral gene expression is largely suppressed, although the limited expression of the early viral genes E1, E2, E5, and especially E6 and E7, results in enhanced proliferation of the infected cells and in their lateral expansion [33]. Following entry into the suprabasal layers, expression of the late viral genes E4, L1, and L2 is initiated; the circular viral genome is then replicated and structural proteins form. In the upper layers of the epidermis or mucosa, complete viral particles are assembled and released [33]. The HPV E1 and E2 proteins are involved in replication and segregation of episomal viral genome [34]; the E2 protein also acts as a transcriptional activator of viral promoters through its ability to bind to specific sequences within the regulatory sequences of HPV genome, known as long control region (LCR) [35]. However, in some cases, E2 may also inhibit viral gene transcription: in genital tract-associated high-risk HPV, E2 protein has been demonstrated to suppress E6 and E7 expression probably through steric interference with the binding of positively acting cellular transcription factors in the LCR [36]. HPV E4 function is probably to aid the virus in its egress from the cell [37]. E5 seems to be important in the early course of infection. It probably stimulates cell growth by forming a complex with the epidermal growth factor receptor or a subunit of the vacuolar ATPase, thus inhibiting the acidification of endosomes and growth factor receptor recycling [38,39]. However, since the E5 gene is not expressed in most HPV-positive cancers, E5 does not seem to be obligatory in late events of HPV-mediated carcinogenesis [33]. A more significant role for malignant transformation can be assigned to the E6 and E7 genes and their respective proteins. They are consistently expressed in malignant tissue, and inhibiting their expression blocks the malignant phenotype of cervical cancer cells [40]. They are independently able to immortalize various human cell types in tissue culture, and show synergistic activity when they are expressed together. Interestingly, in high risk HPVs, E6 and E7 protein are translated together from a polycistronic mRNA [33].

The E7 protein encoded by high-risk HPV is a small multifunctional nuclear protein that binds zinc, is phosphorylated by casein kinase II, and shares structural similarities with oncoproteins of other DNA tumor viruses [41]. The most significant cellular targets of E7 are the retinoblastoma (Rb) tumor suppressor protein and its functionally related proteins, p107 and p130, especially in their hypophosphorylated active forms, which are able to inhibit cell cycle progression by binding and repressing the activity of E2F cellular transcription factors [42]. Binding with high-risk E7 results in pRb proteasomal degradation, but this event is not sufficient to account for E7 transforming functions [43]. Indeed, E7 has additional cellular targets, such as the cyclin-dependent kinase inhibitors p27^kip1 ^and p21^cip1^, which are inactivated [44-46], and causes genomic instability leading to chromosomal abnormalities and aneuoploidy [47].

The first transforming activity identified for the high-risk HPV E6 proteins was the ability to mediate p53 degradation through the ubiquitin proteolysis pathway [48], a property not possessed by the low-risk HPV E6 proteins. E6 first associates with the cellular ubiquitin ligase protein termed *E6-associated protein *(E6AP), then the E6-E6AP complex binds to p53, stimulating its ubiquitination and degradation [49]. In targeting p53, the high-risk HPV E6 proteins inhibit DNA damage and oncogene-mediated cell death signals. E6 from high-risk HPV also interacts with several other cellular proteins involved in DNA replication, signal transduction (NFX1), activation of telomerase (Myc), cell proliferation (PDZ motif-containing proteins), and inhibition of apoptosis (Bak, CBP/p300) [33].

Invasive cervical cancers and precancerous lesions associated with high-risk HPV infection are characterized by integration of HPV DNA in cellular chromosomes. Viral DNA integration is associated with deletion of large segments of viral genome, but with the presence of intact E6 and E7, and with transcription of sequence downstream from the integrated LCR [50]. Disruption of the viral E1 and E2 genes, as well as of downstream viral sequences, may permit higher levels of E6 and E7 transcription, whose RNA are stabilized following fusion with downstream cellular sequences [51]. Persistent expression of E6 and E7 appears to be necessary to maintain the malignant phenotype and to favour the occurrence of cellular genetic and epigenetic events, which are important for cancer progression [52].

## Screening for cervical cancer and diagnosis of HPV infection

Screening programs for cervical cancer prevention derive from knowledge on the natural history of cervical cancer and its precancerous lesions and the role of HPV infection in cancer development. These programs, which vary widely by country, include screening tests for early detection of cancer, as well as management strategies for treatment and post-treatment follow-up. Several international and national scientific societies have drawn guidelines on cervical cancer screening and prevention, which are summarized in Table 1. All these guidelines emphasize the utility to incorporate HPV testing as an adjunct to cervical cytology in cervical cancer screening programs in women aged ≥ 30 years and for management of women with an abnormal Pap test.

**Table 1:** European and US guidelines on cervical cancer screening and prevention.

	**European guidelines for quality assurance in cervical cancer screening**; 2007 [58]	**ACS (American Cancer Society); **2007 [53,54]	**ACOG (American College of Obstetricians & Gynecologists); **2003	**ASCCP (American Society for Colposcopy & Cervical Pathology); **2006 [55,56]	**US Preventive Service Task Force**; 2003
Initiation of screening with Pap cytology	20–30 yr	about 3 yr after the onset of sexual activity but no later than age 21 yr	about 3 yr after the onset of sexual activity but no later than age 21 yr	NR	within 3 yr of onset of sexual activity or age 21 yr, whichever comes first
Use of HPV testing in screening programs	Not recommended while waiting for the results of randomized controlled trials.	With cytology in women ≥ 30 yr		With cytology in women ≥ 30 yr	Insufficient evidence
Screening intervals					
- conventional Pap test	3–5 yr	Annually; every 2–3 yr for women aged ≥ 30 yr with 3 consecutive negative cytology tests.	Annually; every 2–3 yr for women aged ≥ 30 yr with 3 consecutive negative cytology tests.	NR	At least every 3 yr
- if HPV testing used	NR	Every 3 yr if HPV negative and cytology negative.	Every 3 yr if HPV negative and cytology negative.	NR	Insufficient evidence
Discontinuation of screening	60–65 yr with ≥ 3 recent consecutive negative tests.	Women aged ≥ 70 yr with ≥ 3 recent consecutive negative tests and no abnormal tests in prior 10 yr.	Inconclusive evidence to establish upper age limit.	NR	Women aged ≥ 65 yr with negative tests, who are not otherwise at high risk for cervical cancer.
Management of abnormal cervical cancer screening test - ASC-US - ASC-H - LSIL - HSIL	ASC-US: reflex HPV testing; LSIL: repeat cytology or colposcopy; ASC-H: colposcopy; HSIL: colposcopy and biopsy.	NR	NR	ASC-US: HPV testing, or repeat cytology, or colposcopy in women ≥ 20 yr; ASC-H: colposcopy LSIL: colposcopy HSIL: immediate loop electrosurgical excision or colposcopy with endocervical assessment.	NR

NR: not reported.

Guidelines of the American Cancer Society [53,54] recommend that screening should begin approximately 3 years after a woman starts having vaginal intercourse, but no later than age 21 years. Screening should be done every year with conventional Pap tests or every 2 years using liquid-based cytology testing. At or after age 30 years, women who have had 3 normal test results in a row may get screened every 2 to 3 years with cervical cytology alone, or every 3 years with cytology test plus high-risk HPV DNA testing (HPV test). Women aged ≥ 70 years who have had 3 or more normal Pap tests and no abnormal Pap tests in the last 10 years and women who have had total hysterectomy may choose to stop cervical cancer screening. Special recommendations are given to at-risk categories (i.e., history of cervical cancer, in utero exposure to diethylstilbestrol, immunosuppression). Similar guidelines have been proposed by the American College of Obstetricians and Gynecologists in 2003 , by the U.S. Preventive Services Task Force in 2003 , and, more recently, in 2006, by the American Society for Colposcopy and Cervical Pathology [55,56].

In European Union member states, screening programs for cervical cancer prevention are organized according to the European Council [57] and the European Guidelines [58], which recommend to set up cancer screening as a population-based public health programme, with identification and personal invitation of each woman in the eligible target population, and to discourage opportunistic screening. According to these guidelines, cervical cytology is the currently recommended standard test for cervical cancer screening, which should start in the age range 20–30 years. Screening is recommended to be continued at 3–5-year intervals until the age of 60–65. In older women, who have had three or more consecutive previous recent normal cytology results, stopping screening is considered appropriate, while special attention has to be paid to those who have never attended screening. While waiting for the results of ongoing longitudinal trials before recommending HPV screening, HPV DNA testing is proposed in: 1) primary screening for oncogenic HPV types alone or in combination with cytology; 2) triage of women with equivocal cytological results; 3) follow-up of women treated for CIN to predict success or failure of treatment. Referral for colposcopy is recommended for women with a high-grade cytological lesion (ASC-H and HSIL or higher), a repeated low-grade lesion, or with an equivocal cytology result and a positive HPV test. Management options in case of atypical squamous cells of undetermined significance (ASC-US) include reflex HPV DNA testing (preferred option when HPV testing is available), repeating the Pap smear after 6 to 12 months, or referral for colposcopy and cervical biopsy (when poor follow-up compliance is suspected or when explicit risk factors are present). Repeat cytology or referral for colposcopy (preferred option) are considered acceptable options for initial management of LSIL, while HPV testing is considered not sufficiently selective. The guidelines of the Italian Ministry of Health for the cervical cancer screening suggest performing a Pap test every three years in women aged 25–64, as first level of screening, followed by specific recommendations for women with abnormal cytology [59]. In particular, referral for colposcopy is recommended in the case of ASC-H and HSIL or higher at Pap test, while the following management options are acceptable in the case of ASC-US: triage with reflex HPV DNA test (and referral to colposcopy if HPV DNA test is positive), immediate referral to colposcopy, or repeat Pap cytology after 6 months. Referral to colposcopy is the preferred management strategy for LSIL, although triage with HPV testing is also acceptable [59].

### Cytology testing

Screening programs based on cytology testing led to the reduction of the incidence of cervical cancer and cancer-related mortality of about 70–80% in many industrialized countries [1].

The efficacy of conventional cytological screening for cervical cancer has never been investigated in randomised clinical trials, but evidence of its effectiveness derives from observational studies. Diagnostic performance of cervical cytology has been evaluated by several studies in the literature, whose results have been reviewed in recent meta-analyses. These studies have demonstrated that a single cytology test has a sensitivity of about 50% for identifying women with high-grade precancerous lesions or invasive cervical carcinoma (i.e., cervical intraepithelial neoplasia type 2 or higher, CIN2+) [60-63]. In particular, a recent meta-analysis on 8 cervical cancer screening studies done in North American and European countries with established cytology-based screening activities, including over 60,000 women who were tested for both HPV DNA and cytology, found that the overall relative sensitivity of cytology for CIN2+ was 53.0% (95% CI, 48.6%–57.4%), by using ASC-US as cut-off for a positive result [62]. Moreover, sensitivity of cytology varied considerably among studies, ranging from 18.6% to 76.7% [62]. In fact, cytology testing has been shown to have poor inter-laboratory and inter-operator reproducibility [64]. The meta-analysis also showed the sensitivity of cytology testing increases with patient's age and is significantly higher in women over the age of 50 than in younger women (79.3% *vs*. 59.6%, respectively). The overall specificity of cytology for CIN2+ is very high (96.3%; 95% CI, 96.1–96.5%), and this is an important requisite for a screening test, and is even higher in women aged ≥ 35 years than in younger women (97.1% *vs*. 95.9%, respectively) [62]. More recent meta-analyses involving more European/North American studies yielded a pooled sensitivity of cytology at cut-off ASCUS+ for finding CIN2+ of 70% (95% CI: 60–83%). After omission of three German studies, which reported very low sensitivity data, the pooled sensitivity increased to 78% (95% CI: 69–87%) [63,65].

Liquid-based cytology has been more recently introduced and is now widely used for primary screening of cervical cancer. It has the advantage of allowing automation, faster reading times, and to be used for adjunctive testing, including HPV testing. It has been suggested to be more sensitive than conventional cytology, but this feature has been rebutted by recent randomized studies, which compared the accuracy of liquid-based cytology with conventional cytology for primary screening of cervical carcinoma [66,67]. In these studies, liquid-based cytology showed no significant difference in sensitivity to conventional cytology for detection of CIN2+, besides a lower positive predictive value (PPV) due to a higher number of positive results. It showed however the advantage of a reduction of unsatisfactory smears [67].

### HPV test

Currently used molecular tests for the detection of nucleic acids of high-risk HPV in clinical specimens are based on signal-amplification and hybridization methods, such as the Hybrid Capture^® ^II assay (HC2, Diagene Corp., Gaithsburg, Maryland, USA), or on target amplification, like the polymerase chain reaction (PCR)-based Amplicor^® ^HPV test (Roche Molecular Diagnostics, Switzerland) or other PCR-based assays. The HC2 test, to date, is the only commercially available HPV DNA detection test that is approved by the FDA for cervical cancer screening in combination with cytology after the age of 30 years. The HC2 test is a nucleic acid hybridization assay with signal amplification for the qualitative detection of HPV DNA of 13 high-risk types in cervical specimens. The HC2 assay cannot identify the specific HPV type, since detection is performed with a combined probe mix, and provides a semi-quantitative estimate of HPV DNA load. PCR-based assays use consensus or general primers, such as the GP5+/6+ PCR system, the Roche Amplicor^® ^system, the PGMY primer set, and the SPF_10 _primer set, to amplify a broad spectrum of HPV types. These primers target the L1 gene in a conserved region amongst HPVs. Subsequent to amplification of HPV DNA, detection of high-risk HPV types is performed by hybridization with probes specific for high-risk HPVs. HPV genotyping is performed by type-specific PCR or by amplification of HPV DNA by using consensus or general primers, followed by sequencing, restriction fragment length polymorphism analysis, or reverse hybridization of the amplicon to multiple oligonucleotide probes corresponding to high-risk and low-risk HPVs. Reverse hybridization assays, such as the linear array by Roche Diagnostics, the line probe assay by Innogenetics NV (Belgium), microchips by Biomedlab Co. (Korea) and by other companies, Multiplexed Luminex Assay (Multimetrix, Germany), etc., are able to identify co-infections by different HPV types, at variance with sequencing-based assays, which have high accuracy in the definition of HPV types but poor sensitivity in co-infections.

## Applications of HPV testing in cervical cancer screening programs

### Using HPV DNA test for triage of abnormal Pap test

HPV testing has been introduced in programs for cervical cancer prevention after numerous studies had demonstrated that detection of high-risk HPVs has a higher sensitivity than cytology testing in predicting high-grade cervical precancerous lesions or invasive cervical carcinoma. A meta-analysis on the use of HPV testing to manage women with ASC-US showed that HPV testing has a greater accuracy than repeat cervical cytology [61]. In fact, the overall sensitivity and specificity of HPV testing were 84.4% and 72.9%, respectively, *vs*. 57.6% and 81.8% of Pap-test, by using ASC-US as cut-off for a positive result; by using LSIL as cut-off, Pap test sensitivity was reduced to 45.7%, whereas specificity increased to 89.1% [61]. Among the studies included in the meta-analysis, there was the large ALTS (ASCUS Low SIL Triage Study), a controlled clinical trial, which enrolled 3,488 women, randomized into three intervention groups: immediate colposcopy, repeated liquid-based cytology, or HPV testing. This trial, as confirmed also by other similar studies, demonstrated that HPV testing for ASC-US triage had a significantly higher sensitivity and similar specificity for CIN3+ than repeat cytology [68,69]. Based on these results, both the American Society for Colposcopy and Cervical Pathology (ASCCP) [55,56] and the European Guidelines [58] currently recommend HPV testing using validated HPV assays as an acceptable method for managing women over the age of 20 years with ASC-US. According to ASCCP guidelines, women with ASC-US who are HPV DNA negative can be followed up with repeat cytological testing at 12 months, whereas those who are HPV DNA positive should be referred for colposcopic evaluation. If CIN is not identified, repeat HPV testing at 12 months is recommended. However, women with ASC-US who are HPV DNA negative have a very low probability of having CIN2+, lower than the general population with negative cytology and unknown HPV status, who is actually referred to three years. So, a more conservative management strategy than ASCCP recommendations could be appropriate.

HPV testing has not been demonstrated to be a useful management option for women with a cytological result of LSIL. In fact, a meta-analysis of published studies demonstrated HPV triage of LSIL had no significantly higher sensitivity but lower specificity than repeat cytology in the detection of high-grade CIN [70]. In particular, the ALTS study found that over 80% of women with LSIL were HPV-positive, whereas the prevalence of CIN2+ was considerably lower [71]. Nevertheless, reflex HPV testing may be cost effective in older women with LSIL, due to considerably lower prevalence of HPV infection. In this regard, data from the randomized Italian study New Technologies for Cervical Cancer Screening (NTCC), which compared accuracy of cytology testing *vs*. HPV DNA testing, specificity of HPV testing for triage of LSIL was higher in women aged over 35 years than in younger women, since the proportion of HPV positive women was much lower in women aged 35–60 years than in those aged 25–34 years (42% *vs*. 72%) [72]. So, HPV triage could be appropriate also for LSIL in women over 35 years, in order to reduce colposcopy burden and to increase the positive predictive value of the colposcopy referral.

### Using HPV DNA test for follow-up after treatment of cervical dysplasia

Several studies demonstrated that HPV testing, performed at 4–6 months intervals following ablative therapy for high-grade lesions (CIN2–3), has a higher sensitivity and specificity than cytology in detecting residual disease or recurrence [73-75]. In a meta-analysis of 11 studies evaluating HPV DNA testing in monitoring women after treatment of CIN3, the negative predictive value of HPV testing for recurrent/residual disease was 98%, whereas the negative predictive value of cytology was 93% [76]. Overall, the sensitivity of HPV testing for identifying recurrent/persistent CIN reaches 90% by 6 months after treatment and remains high for at least 24 months, at variance with cytological follow-up, which has a sensitivity of approximately 70%. These findings were confirmed in an updated meta-analysis, which showed HPV testing has a significantly higher sensitivity (ratio: 1.27; 95% CI: 1.06–1.51) and not-significantly lower specificity (ratio: 0.94; 95% CI: 0.87–1.01) than follow-up cytology in the prediction of residual/recurrent CIN [65].

HPV testing at 6–12 months for post treatment management of CIN2,3 is recommended by current guidelines of the American Society for Colposcopy and Cervical Pathology [56], whereas European Guidelines, while waiting for further data from clinical trials, recommend double testing with cytology and an HPV test at 6 months post treatment [58].

### Using HPV DNA test for cervical cancer screening

The potential role of HPV testing as a stand-alone test or in conjunction with cytology in cervical cancer screening has been investigated in several studies [reviewed in [62,65,77]]. Unfortunately, these studies were performed with different techniques and sometimes gave conflicting results.

A meta-analysis including 25 non-randomized clinical trials, which compared the performance of cytology vs. HPV test in screening programs for prediction of CIN2+ [63], showed the overall sensitivity of HPV test was 90%, when the HC2 assay was used, and 80.9%, when PCR with consensus primers was employed; overall specificity was higher in PCR-based tests than in HC2, being 94.7% and 86.5%, respectively. The accuracy of cytology testing was lower than HPV testing: in fact, with ASC-US as a cut-off, sensitivity was 72.7% and specificity 91.9%. In women aged over 30 yr, the overall sensitivity of the HC2 assay was 94.8% and specificity 86% [63].

Likewise, the meta-analysis by Cuzick *et al*. [62], which included only studies from European and North American countries, where screening programs are well implemented, showed the overall sensitivity and specificity of HPV testing were 96.1% and 90.7%, respectively, whereas the overall sensitivity and specificity of cytology testing were 53.0% and 96.3%, respectively. Moreover, at variance with cytology testing, sensitivity of the HPV test did not vary among the different Centres and was very high both in young women and in those over 50 years of age. Among the studies included in this meta-analysis, there was the HART (HPV in Addition to Routine Testing) trial, a multicentric screening protocol which enrolled over 10,000 women aged 30–60 years attending for routine screening. In this study, women with borderline cytology or negative cytology but positive HPV test were randomized to immediate colposcopy or follow-up with repeat HPV test, cytology testing, or colposcopy at 12 months [78]. The HART study confirmed HPV testing is more sensitive than borderline or worse cytology (97.1%, 95% CI 91.2–99.1%, *vs*. 76.6%, 95% CI 65.1–85.1%) but less specific (93.3%, 95% CI 92.7–93.9%, *vs*. 95.8%, 95% CI 95.4–96.2%) for detecting CIN2+ [78]. Comparison of management strategies for borderline cytology and for positive HPV test with negative cytology results at baseline showed that surveillance at 12 months was as effective as immediate colposcopy [78]. Interestingly, no CIN2+ were detected in women with a positive HPV test at baseline but negative at follow-up nor in those with an initial negative HPV test and borderline or mild cytology [78]. Based on these results, it was suggested that HPV test could be used as a screening test in women over 30 years of age with cytology triage of HPV-positive results. In case of normal or borderline cytology, repeat HPV testing after 12 months was indicated [78].

Randomized longitudinal studies [79], which have been conducted more recently, were not included in the meta-analyses. One of these studies is the NTCC study, an ongoing clinical trial in Italy nested in the routine activity of organized screening programmes, which enrolled approximately 95000 women, who were randomly assigned to conventional cytology or to an experimental arm that followed two phases depending on the period of recruitment [23]. During the phase 1 of recruitment, women were randomly assigned to the conventional arm for screening by conventional cytology or to the experimental arm, which used both HPV DNA testing with HC2 and liquid-based cytology. HPV-positive women were managed differently according to age. Among women aged 35–60 years, those who had a cytological abnormality or were HPV-positive were referred to colposcopy. Among women aged 25–34 years, all women with abnormal cytology were referred for colposcopy but those who were HPV-positive with normal cytology were retested after 1 year and referred for colposcopy only if HPV positivity persisted or if cytology became ASC-US+. During the phase 2 of recruitment, women were randomly assigned to conventional cytology with referral for colposcopy if cytology indicated ASC-US+ or to HPV DNA testing alone with referral for colposcopy if the test was positive [23]. Results at recruitment showed that the screening method in the experimental arm was more sensitive than conventional cytology in women aged 35–60 years [80]. In particular, referral based on HPV testing with HC2 at a high cut-off value of 2 pg/mL showed a higher sensitivity than conventional cytology for detection of CIN2+ (relative sensitivity, 1.41%; 95% CI, 0.98–2.01), but a lower positive predictive value (PPV; relative PPV, 0.75; 95% CI, 0.45–1.27). The use of the 2 pg/mL cut-off led to a higher PPV than the 1 pg/mL cut-off [80]. Adding liquid-based cytology improved sensitivity only marginally but increased false positives [80]. In women younger than 35 years, HPV testing alone as primary test, with triage of HPV-positive women by cytology, appeared to be the best approach, since the use of liquid-based cytology did not increased the sensitivity for CIN2+ detection and had a significantly lower PPV as compared with conventional cytology. Moreover, addition of liquid-based cytology as a primary screening test to HPV testing had a negligible effect on sensitivity but strongly reduced PPV compared with HPV testing only [81]. The use of more restricted referral criteria (i.e., cut-off of 2 pg/mL and positive results at repeat HPV testing) was predicted to increase PPV to that obtained with conventional cytology [81].

Results from the second recruitment phase of the NTCC study showed that, in women aged 35–60 years, HPV testing alone with a cut-off of 2 pg/mL achieved a substantial gain in sensitivity over conventional cytology with only a small reduction in PPV (relative sensitivity, 1.81; 95% CI, 1.20–2.72; relative PPV, 0.99; 95% CI, 0.67–1.46) [23], confirming the results of the first phase of the study [80]. In women aged 25–34 years, direct referral to colposcopy after a positive HPV test led to detection of a significantly greater number of CIN2+ and CIN3+ lesions than in the first phase of the study [23]. The increased sensitivity of HPV testing for CIN2+ in the second phase of the study as compared with the first was suggested to be related to a high rate of high-grade lesions in young women, which spontaneously regress [23]. So, in order to avoid overtreatment, it was suggested that HPV-positive women aged 25–34 years should be referred to colposcopy only if cytology is also abnormal or if infection persists after 1 year [23]. In older women, high-grade lesions are less likely to regress [78,80], therefore a more aggressive management strategy for HPV-positive results appears to be justified. On the other hand, referral to colposcopy of women with abnormal cytological findings (ASC-US+) but a negative HPV test may led to a high-rate of false positive CIN2+ results and, as a consequence, to overtreatment [82].

Persistence of the additional lesions detected at baseline by HPV testing in older women was also suggested by the recently published results from two longitudinal randomized controlled trials, that included only women at least 30 [83] or 32 [84] years of age. One of the trials is the Population Based Screening Study Amsterdam (POBASCAM), which aims to assess whether primary HPV DNA testing is more effective than cytological testing in the setting of a regular screening programme. This study enrolled women aged 30–56 years and attending the regular cervical cancer screening programme in the Netherlands. Women were randomly assigned to the intervention group (screening with HPV DNA testing combined with Pap test) or control group (Pap test only, HPV DNA test results blinded). After 5 years, combined cytological and HPV DNA testing were done in both groups and follow-up data for ≥ 6.5 years were available for 17,155 women. Management strategies were more conservative than in the NTCC study. In fact, women with HSIL or worse were immediately referred to colposcopy, irrespective of the HPV result, whereas repeat testing after 6 and 18 months was advised to women with normal cytology but positive HPV test and to those with ASC-US, ASC-H, or LSIL. Women with repeat positive cytology and HPV test results were referred to colposcopy [83]. In this study, detection of HPV DNA was done by GP5+/6+ PCR followed by enzyme immunoassay detection of 14 high-risk HPV types with a cocktail of oligonucleotide probes. The other trial was conducted in Sweden and enrolled 12,527 women aged 32 to 38 years, who, like in the POBASCAM study, were randomized to have an HPV test plus Pap test or a Pap test alone [84]. In this study, HPV test was based on GP5+/6+ PCR followed by reverse dot blot hybridization to identify 14 high-risk HPVs. Women with a positive HPV test and a normal Pap test were offered a second HPV test at least 1 year later, and those who were found to be infected with the same high-risk HPV type were offered colposcopy with cervical biopsy. To avoid ascertainment bias, a similar number of Pap smears and colposcopy with biopsies were performed in randomly selected women in the control group. All women were followed for a mean of 4.1 years with annual Pap smears and HPV tests, with colposcopy in cases of persistent high-risk HPV infection [84]. Both studies showed that a significantly higher number of CIN2+ or CIN3+ lesions were detected at baseline in the intervention group than in the control group, whereas the number of lesions detected in the subsequent follow-up visit, when both HPV test and cytology were done, was significantly lower in the intervention group than in the control group [83,84], thus indicating that implementation of HPV DNA testing in cervical cancer screening leads to earlier detection of high-grade lesions, thus allowing longer screening intervals. In the Dutch study, the 5-year cumulative risk of CIN3+ was 0.1% (95% CI, 0.1–0.2%) after a combined negative HPV DNA and cytological result at baseline and 0.8% (95% CI, 0.6–1.0%) after a negative cytology at baseline, but without HPV testing [83]. After a negative HPV test at baseline, the 5-year cumulative risk of CIN3+ was estimated as 0.2% (95% CI, 0.1–0.3%) [83]. Likewise, in a longitudinal cohort study which enrolled over 20,000 women in Portland who underwent simultaneous screening with a Pap test and HPV testing with HC2, the 5-year cumulative risk of CIN3, was 4.4% for women who were HPV-positive at baseline, but only 0.24% among women with a negative HPV test and 0.16% when both the HPV test and Pap smear were negative [85].

A direct comparison between Pap test and HPV test (by using the HC2 assay) as stand alone tests for cervical cancer screening was evaluated in a recent randomized trial in Canada, involving 10,154 women aged 30–69 years [86]. Both tests were performed on all women in a randomly assigned order but, to the aim of the study, only one first test was considered for statistical evaluation. Women with abnormal Pap test results or a positive HPV test at 1 pg/mL cut-off underwent colposcopy and biopsy, as a random sample of women with negative tests. Results at baseline testing showed sensitivity of HPV testing for CIN2 or CIN3 was 94.6% (95% CI, 84.2–100%), significantly higher than sensitivity of Pap test, which was 55.4% (95% CI, 33.6–77.2%). HPV test specificity was 94.1% (95% CI, 93.4–94.8%) and 96.8% for Pap testing (95% CI, 96.3–97.3%) [86].

**Recommendations on the use of HPV testing in cervical cancer screening programs **(grading of recommendations is reported in Table 2)

**Table 2:** Key to evidence statements and grading recommendations defined by SIV.*

***Strength of Recommendation***
A. Good evidence for efficacy and substantial clinical benefit support recommendation for use.
B. Moderate evidence for efficacy or only limited clinical benefit supports recommendation for use.
C. Evidence for efficacy is conflicting and does not allow supporting a recommendation for or against use, but recommendations may be made on other grounds.
D. Moderate evidence for lack of efficacy or for adverse outcome supports a recommendation against use.
E. Good evidence for lack of efficacy or for adverse outcome supports a recommendation against use.
I. There is insufficient evidence (in quality and quantity) to make a recommendation; however, other factors may influence decision-making.

***Quality of Evidence***
I. Evidence from at least 1 randomized, controlled trial.
II. Evidence from at least 1 clinical trial without randomization, from cohort or case-controlled analytic studies (preferably from more than 1 centre) or from multiple time-series studies or dramatic results from uncontrolled experiments.
III. Evidence from opinions of respected authorities based on clinical experience, descriptive studies, or reports of expert committees.

*: Quality of evidence and grading of recommendations were defined and approved by SIV; they were adapted from The Evaluation of Evidence and the Classification of Recommendations criteria described in The Canadian Task Force on Preventive Health Care.

• HPV testing is recommended for ASC-US triage since HPV test is more sensitive than repeat cytology for detection of CIN2+ (IA).

• HPV testing is, in general, not a management option in case of LSIL (ID), even if it may be cost effective in older women with LSIL (IB). Local assess is needed to identify a good triage test for women with LSIL (research need).

• HPV testing, alone or in combination with cytology, is recommended for follow-up after treatment of high-grade cervical lesions, since it is more accurate than repeat cytology in diagnosing residual disease or relapse (IA).

• HPV testing, both based on hybridization (HC2) and PCR amplification with detection of high-risk HPVs, has been widely used in women aged >30 years in addition to Pap cytology because of the high NPV of a combined negative test (I). Women with normal Pap test result and negative HPV test have a very low risk to develop cervical cancer and should not perform the subsequent follow-up visit earlier than 5 years (IA). However, combination of HPV testing and Pap cytology is not a cost-effective strategy (I), so, strategies based on HPV testing as a stand alone test should be preferred in women aged >30 years (IA).

• Follow-up with HPV testing or repeat cytology at 12 months is recommended for cytology-negative, HPV-positive women aged ≥ 30 years (IA). Women who are persistently HPV-positive on repeat testing should undergo colposcopy, whereas those who are negative on both tests may be rescreened in 5 years (IA).

• Using the HPV test in screening programs leads to earlier detection of CIN2+ and CIN3+ and reduction of the number of high-grade lesions detected at subsequent follow-up in women aged ≥ 30 years (I), thus allowing longer screening intervals. The extent of long-term protection against high-grade lesions after a negative HPV DNA test has been estimated to be longer than 5 years, but further research is need to establish the most appropriate screening interval (research need).

• HPV testing as a stand alone test is more effective than cytology alone in identification of CIN2+ lesions in women aged ≥ 35 years (I) and might be used as a primary screening test in this age category. First follow-up results from ongoing controlled randomized trials, which compare longitudinal performance of Pap test *vs*. HPV testing in screening programs, confirm the higher efficacy of HPV screening that Pap screening (I). Local assessment is needed before screening policies based on primary HPV testing can be recommended (research need).

• In women aged < 35 years, HPV testing with immediate referral to colposcopy leads to over diagnosis of CIN2+ as compared with more conservative management strategies (I). Thus, in women younger than 35 years of age with a positive HPV test, cytology triage or repeat HPV testing at 12 months could be appropriate management strategies to avoid over diagnosis and over treatment of CIN2+, but this hypothesis needs confirmation in longitudinal studies (research need).

## New molecular tests for management of women with HPV infection

Due to the relatively low specificity of HPV-DNA testing and the high rate of false negative results at Pap test, new molecular tests have been proposed in order to identify women with a higher risk of progression to invasive cancer. These tests include (i) detection of type-specific HPV persistence; (ii) detection of high-risk HPV E6/E7 mRNAs; (iii) HPV genotyping to detect HPV-16 and HPV-18, which have been associated with a higher risk of CIN2,3 or invasive cancer; (iv) measurement of high-risk HPV load; (v) demonstration of high-risk HPV DNA integration; (vi) HPV subtyping by E6, L1, and LCR sequencing; (vii) analysis of p16-INK4A expression. The implementation of these new molecular tests in cervical cancer screening could be useful to identify the subpopulation of high-risk HPV-positive women who deserve a closer follow-up and appropriate treatment.

### Demonstration of HPV persistence

In women younger than 25 years of age, 20% of high-risk HPV infections persist, whereas the risk of persistent infection is over 50% in women older than 55 years [18]. Only a few women with HPV infection will develop cancer. Thus, a single positive HPV test in the absence of clinically significant lesions does not justify treatment [87]. Several longitudinal studies demonstrated that persistence of high-risk HPV is necessary for cancer initiation and progression and that the majority of high-risk HPV infections and associated low-grade lesions spontaneously regress within 6–18 months [1,88-93]. Screening strategies based on repeat HPV testing at 12 months following detection of a high-risk HPV could be useful to identify women at risk of cancer, who should undergo colposcopy, and to avoid overtreatment of lesions which will regress [94,95].

From a practical point of view, persistence can be defined as the detection of the same HPV type (or, with a higher degree of certainty, the same intratypic variant) two or more times over a certain period. There is no consensus as to the length of time that implies persistence, but at least 6 months to 1 year is the time frame that is usually chosen [1]. A prospective study of type-specific HPV natural history, with a median follow-up of 5.1 years, showed particularly pronounced persistence of HPV-16 and markedly increased risk of CIN3+ occurrence when HPV-16 persisted, compared with any other HPV type [96]. Evaluation of type-specific HPV persistence was used as a management strategy for women with a positive HPV test but normal Pap test in a controlled randomized trial in Sweden, as above reported [84].

### Analysis of high-risk HPV E6/E7 mRNA

Cervical cancer is characterized by overexpression of E6/E7 mRNA of high-risk HPVs. A multiplex nucleic acid sequence based amplification system (NASBA) assay was developed for the identification of E6/E7 mRNA from HPV types 16, 18, 31, 33, and 45 (PreTect™ HPV Proofer kit, NorChip AS, Norway; now also distributed as NucliSENS EasyQ^® ^HPV by bioMérieux SA, Marcy l'Etoile, France). Analysis of E6/E7 mRNA expression with the PreTect™ HPV Proofer kit in 204 histologically confirmed invasive cervical squamous cell carcinomas from Norwegian women demonstrated the presence of oncogenic transcripts of HPV-16, HPV-18, HPV-31, HPV-33, and HPV-45 in 97% of HPV-positive cases, thus confirming the expression of oncogenic E6/E7 in most invasive cancers and the involvement of the above HPV types in most cases [97]. A cross-sectional study was performed in 4,136 women >30 years of age to compare the performance of HPV E6/E7 mRNA detection with detection of HPV-DNA by Gp5+/6+ consensus PCR [98]. No significant difference in sensitivity and specificity was observed between the two tests for detection of HPV in high-grade squamous intraepithelial lesion (HSIL). On the contrary, only a small proportion of the HPV DNA-positive women with a normal, ASC-US, or LSIL diagnosis had a detectable E6/E7 mRNA expression [98]. A comparative study between HPV testing with Gp5+/6+ consensus PCR and high-risk HPV E6/E7 mRNA testing with the PreTect™ HPV Proofer kit showed the latest test had equal sensitivity and significantly higher specificity for CIN2+ than PCR [99]. Moreover, E6/E7 mRNA-positive women had a significantly higher risk to have a CIN2+ within 2 years than negative women [99]. Interestingly, quantitative analysis of E6/E7 mRNA in cervical cancers, but not HPV DNA load, correlated with patient survival [100]. The APTIMA HPV^® ^Assay, developed by Gen-Probe Incorporation (San Diego, CA), employs transcription-mediated amplification and target capture to detect HPV E6/E7 mRNA from 14 high-risk HPV types, but it does not differentiate among the 14 high-risk types. Preliminary results obtained with this assay suggest it has higher sensitivity and specificity than HPV testing for identification of CIN2+ lesions [101].

### Measurement of viral load

As above reported, higher cut-off values improve the specificity of the HC2 test [80-82] and correlate with an increased risk of HPV persistence and development of high-grade cervical lesions [78]. A more accurate quantification of viral load can be achieved by using real-time PCR to detect specific HPV types. This method allowed demonstrating that high HPV-16 DNA load is associated with CIN3 and invasive cervical carcinoma or with an increased risk to develop CIN2–3 during follow-up [92,102-106]. Thus, measurement of HPV-16 load by real-time quantitative PCR has been proposed as a marker of risk for CIN progression. Contrasting results have been obtained with other high-risk HPV types, although, generally, a positive relationship between viral load and severity of cervical lesions has been reported [107-109].

### HPV typing and subtyping

Women with HPV-16 and HPV-18 infection have a higher probability to develop cancer than women with infection by other high-risk HPV types [110-113]. Some studies demonstrated that detection of HPV-16 and HPV-18 types represents a risk factor for CIN2–3 [111,112], but also identification of other high-risk HPV types could be useful to select at risk patients [114,115]. Investigation of intra-typic variants of HPV, which are defined by a difference in the L1 gene sequence less than 2% of the reference HPV prototype, have been proven to be useful in epidemiological studies. In multiethnic populations, Asiatic-American and other non-European variants of HPV-16 and HPV-18 are associated with a higher risk of persistent infection and development of cervical precancerous lesions and invasive cancer [116-118].

Investigation of high-risk HPV E6 e E7 variants also gave interesting results. The T350G sequence variant of the HPV16 E6 gene is associated with increased viral persistence and oncogenicity [119,120]. The aminoacid change caused by this mutation could affect E6 degradation activity on p53, besides changing its immunogenicity. Moreover, the L83V mutation of HPV16 E6 increases activation of the MAPK pathway and cooperates with Notch 1 in tumorigenesis [121]. HPV-16 sequence variants may also be associated with different oncogenic potential, even in the absence of virus integration, due to derepression of the E6 and E7 promoter [122,123]. Thus, typing, subtyping, and sequencing of HPV oncogenes and the upstream regulatory region could be useful for epidemiological purposes but also as prognostic markers.

### Number of HPV types included in HPV tests

Demonstration that some new HPV types, defined as intermediate-risk, such as HPV-26, 53, 66, 73, and 82, may be associated with the risk of cervical cancer has suggested to extend the number of HPV types included in HPV DNA tests. To assess the clinical impact of such an extended test, the results from two randomized trials which used HPV test for cervical cancer screening and ASC-US triage, respectively, were analyzed [124]. In the cancer screening protocol, addition of other high-risk HPV types, besides the 12 most common, did not ameliorate test sensitivity. Likewise, when HPV testing was used for ASC-US triage, test sensitivity was the greatest with 17 genotypes, but specificity was very low [124]. Thus, at the moment, increasing the number of high-risk HPV types, besides those already present in the FDA-approved HC2 test, does not seem to be advantageous neither for screening nor for triage of an abnormal Pap smear. On the other hand, reduction of the number of high-risk types included in the HPV test to those most common in invasive cervical cancer (e.g., HPV-16, HPV-31, HPV-33, etc.) could improve HPV test specificity [125].

### Analysis of HPV DNA integration

Integration of HPV-16 and other high-risk HPVs into the genome of infected cells is an important step in tumorigenesis, since it promotes expression of E6/E7 oncogenes. Clinical studies demonstrated HPV DNA integration is associated with a higher risk of treatment failure and with a shorter disease-free interval than cases without integration [126,127]. Thus, assessment of HPV genome integration, by Southern-blot or quantitative real-time PCR analysis of E2/E6 ratio, could represent a good prognostic marker.

### Analysis of p16-INK4A expression

In high-risk HPV infection, E7 binds to and degrades RB which results in substantial up regulation of p16-INK4A synthesis. In spite of the high level of p16-INK4A synthesis, this protein remains functionally inactive, as E7 induces cyclin A and cyclin E expression, thereby functionally bypassing its interference with the cell cycle. Antibodies that are directed against p16-INK4A allow selective staining of high-risk HPV-infected histological sections or of cytological smears, but not other cervical epithelia, suggesting that detection of this marker could provide diagnostic support to distinguish true CIN/dysplasia from immature metaplasia or other non-neoplastic changes of the cervix [128-131] and to improve interpretation of histology [132]. Sensitivity and specificity for CIN2+ of p16-INK4A overexpression testing by immunostaining in cervical cell samples from HPV-DNA-positive women were estimated in a nested sub-study of the NTCC trial [133]. This study demonstrated that HPV testing with p16-INK4A triage improves test specificity for CIN2+. In fact, p16-INK4A triage allows maintaining all the gain in sensitivity obtained by HPV testing alone with respect to conventional cytology, but with referral to colposcopy similar to that of conventional cytology [133].

### Analysis of immune response against HPV

Both antibody-mediated and cell-mediated immune responses are essential for clearance of HPV and HPV-related cervical lesions. The immune response against HPV (and HPV *virus-like particles*, VLPs) is mainly directed against type-specific immunodominant conformational epitopes, although cross-reactivity has been documented, such as between HPV-6 and HPV-11, HPV-31 and HPV-33, HPV-18 and HPV-45. Serum levels of anti-HPV antibodies are stable, even after virus clearance, so HPV serological assays simply demonstrate previous exposure to the virus. Sensitivity of HPV serology is low (50–60%) while specificity is high (about 90%). The low sensitivity of the test is in part explained by lack of seroconversion in some individuals with documented HPV infection [134]. On the other hand, high anti-HPV antibody titre is more common in women with persistent infection than in those who clear infection [134-136]. Moreover, seropositivity for different high-risk HPV types is associated with high-grade CIN [137]. Unfortunately, no standardized and validated HPV serological assays are available [138,139].

Cell-mediated immune response is important for HPV clearance, as suggested by the high incidence of persistent HPV infection in AIDS or transplanted patients. Persistent infection and cancer progression is also associated with abnormal response of CD4+ T cells [140] and CD8+ T cells [141].

#### Evidence synthesis on the use of new tests for diagnosis of HPV infection

• Most high-risk HPV infections recover spontaneously within 6–12 months (I); persistent high-risk HPV infection and high-risk HPV E6/E7 expression are necessary events for cervical cancer development and represent risk factors for cervical cancer (I).

• HPV typing and subtyping provides epidemiological data on HPV distribution and diffusion (II).

• In epidemiological surveys, type-specific HPV detection could contribute to the evaluation of anti-HPV vaccine efficacy (III).

• High HPV DNA load is a risk factor for cervical cancer (II).

• HPV testing with p16-INK4A triage improves test specificity for CIN2+ (II).

• Detection of anti-HPV antibodies demonstrates previous HPV exposure, but this test has low sensitivity (II); a high antibody titre might indicate persistent infection (II).

• Patients with invasive cervical carcinoma have a deficient HPV-specific cell-mediated immune response (II).

#### Recommendations on the use of new tests for diagnosis of HPV infection

• In order to demonstrate HPV persistence, repeat HPV testing should be done at intervals of at least 12 months (IA).

• HPV typing and subtyping in women with a normal or dubious Pap test is useful since it allows to document persistent infection by the same oncogenic HPV type (II) and to demonstrate the presence of HPV-16 and HPV-18 DNA which are markers of increased risk for cervical cancer (II). Women with persistent high-risk HPV infection or with the presence of HPV-16 and HPV-18 DNA should be referred to colposcopy (B).

• Measurement of HPV-16 and other high-risk HPV DNA load could be employed for triage of women with dubious Pap test or with a positive HPV test (B), but further research is needed to identify appropriate management strategies for women with high HPV load (research need).

• Detection of high-risk HPV E6/E7 mRNA could be employed for triage of women with dubious Pap test or with a positive HPV DNA test (IIB). Due to its high sensitivity and specificity (II) HPV E6/E7 mRNA testing should be investigated as a stand alone test in primary screening for cervical cancer (research need).

• HPV typing and subtyping is useful for epidemiological investigation of HPV distribution and diffusion (B).

• Detection of p16-INK4A expression could be used as triage of HPV positive cases (B) and abnormal Pap smears (B) and to confirm histological analysis of cervical biopsies (B).

• Further research is needed to develop standardized methods to measure anti-HPV immune response (research need).

• In epidemiological surveys, evaluation of antibody-mediated and cell-mediated anti-HPV immune response can be used to assess immune response to anti-HPV vaccination and to monitor duration of vaccine protection (B).

## Prophylactic HPV virus-like particle vaccines

Two vaccines targeting HPV-16 and HPV-18, the types that are responsible for 70% of invasive cervical cancers [13,16], have been recently developed and experimented for immunogenicity, safety, and efficacy in randomized clinical trials. These vaccines are based on the self-assembly of recombinant L1 protein into VLPs, that are non-infectious capsids that contain no genetic material. They were first developed and evaluated as monovalent vaccines targeting HPV-16 [142,143], thereafter as a bivalent vaccine targeting HPV-16 and HPV-18 [144-146] and a quadrivalent vaccine against low-risk HPV-6 and HPV-11, besides HPV-16 and HPV-18 [147-149]. Intramuscular injection of the vaccine induces high titres of neutralising antibody in almost all subjects within a month from completion of the vaccination protocol [145,146,148-153].

Two HPV VLP vaccines have been developed and are available for primary vaccination in the European Union: an AS04 adjuvanted L1 VLP vaccine against HPV-16 and HPV-18 (Cervarix^®^, produced by GlaxoSmithKline), which is administered i.m. in three doses (time 0, 1, and 6 months), and a quadrivalent alum adjuvanted L1 VLP vaccine against HPV-16, HPV-18, HPV-6, and HPV-11 (produced by Merck & Co., Inc.; distributed in Europe by Sanofi Pasteur MSD as Gardasil^® ^and by Merck Sharp & Dohme as Silgard^®^), which is administered i.m. in three doses (time 0, 2, and 6 months). The bivalent vaccine is advised for reduction of precancerous cervical lesions and cancer incidence. The quadrivalent vaccine, besides cervical lesions, is advised for reduction of vulvar precancerous lesions and genital condylomas.

The USA and several other Countries are targeting 11–12-year-old girls for vaccination. In particular, the Italian HPV vaccination program is universally offered free-of-charge to all 12-year old girls, and advised to all adolescent women before sexual debut. The choice of a target population of young girls is based on the results of immunogenicity and safety studies, which were conducted in girls and boys aged 9–15 years (quadrivalent vaccine) and aged 10–14 years (bivalent vaccine). These studies demonstrated that both vaccines are safe and immunogenic, and induce antibody titre which is more than 50 times the titres induced by natural infection [145,146,150-156].

### Efficacy of HPV VLP vaccines

Evaluation of vaccine efficacy in phase II-III clinical trials was based on the demonstration of reduction of the incidence of persistent HPV infection (two positive detections at 4–6 months intervals) and on reduction of the incidence of precancerous lesions (CIN2 and CIN3) caused by HPV vaccine types. International standards for treatment of CIN2/3 are ablative therapy. Moreover, in prospective studies, it is not ethical to allow a woman to develop invasive disease in order to demonstrate efficacy of prevention strategies. In this regard, WHO and other international agencies indicated CIN2/3 as clinical endpoint surrogate to demonstrate efficacy of cancer prevention.

Randomised clinical trials demonstrated that three doses vaccination is effective for the prevention of persistent infection and for precancerous lesions caused by HPV genotypes included in the vaccine. Results on efficacy reported in published studies are mainly two types: (i) *per protocol *analysis, which demonstrates the pure efficacy of the vaccine, obtained by analysis, in the experimentation context, of clinical data of women who did not violate the protocol (woman who received three doses of vaccine or placebo as expected), who resulted negative for all HPV genotypes included in the vaccine both at enrolment or during the administration of the vaccine. (ii) *modified intention to treat *analysis, where evaluation of efficacy is more similar to the clinical setting in the general population, since it includes all enrolled women, as long as they receive at least the first dose of vaccine or placebo and who resulted negative for all HPV genotypes of the vaccine merely at the time of administration of first vaccine dose.

#### Efficacy of the quadrivalent HPV VLP vaccine

A phase II randomised trial was carried out on 552 women (277 randomised in the vaccine group and 275 in the placebo group). *Per protocol *efficacy analysis demonstrated that Gardasil^® ^reduced by 90% (95% CI, 71–97%) the incidence of persistent infection and genital lesion associated with the HPV genotypes included in the vaccine [147]. A subset of subjects participated to a subsequent follow-up of this study. Following five years from enrolment, efficacy on combined incidence of persistent infection of HPV-6/11/16/18-related disease was 96% (95% CI 83.8–99.5%) [143]. *Modified intention to treat *analysis demonstrated that vaccination reduced incident infections and cervical lesions by 94% (95% CI, 83–98.3%) [152].

Phase III efficacy studies of the quadrivalent vaccine Gardasil^® ^were conducted in the context of the two protocols Females United to Unilaterally Reduce Endo/Ectocervical Disease (FUTURE) I and FUTURE II:

- FUTURE II trial. *Per protocol *analysis in over 10,000 women (5,305 randomised in the vaccination group and 5,260 randomised in the placebo group) aged 15–26 years demonstrated that, a after a mean 3 year follow-up period, vaccine efficacy was 98% (95% CI, 86–100%) for prevention of CIN2,3 and *in situ *adenocarcimoma associated with HPV-16 and HPV-18 [148]. It is to notice that the unique woman, included in the vaccine group, who developed a HPV-16 or HPV-18 associated lesion, had a positive CIN3 for HPV-52 at the initial control and in all the following 5 samplings during follow-up, only one these samples resulted to be HPV-16 positive. *Modified intention to treat *analysis (definedalso *unrestricted susceptible population*) in randomised women (95% of the study population), who were negative for the vaccine types at the first dose administration, and who did not strictly adhere to the study protocol, demonstrated vaccine efficacy was 95% (95% CI, 85–99%). *Intention to treat *analysis including all randomised subjects, without any pre-vaccine screening, was used to predict efficacy of vaccination on incident/prevalent HPV infections and related lesions in the general population, who demonstrated an impact of 44% (95% CI 26–58%) at 3 years follow-up.

- FUTURE I trial. *Per protocol *analysis on over 5,000 women (2,261 randomised in the vaccination group and 2,279 randomised in the placebo group) aged between 16 and 24 years showed that after an average period of 3 years since vaccine administration, the vaccine had 100% efficacy (95% CI 94–100%) for prevention of condylomas, vaginal and vulvar intraepithelial neoplasia related to HPV-6/11/16/18 and 100% efficacy (95% CI 94–100%) for prevention of CIN1–3 or *in situ *adenocarcinoma associated with vaccine HPV genotypes [149]. Modified intention-to-treat analysis (also defined *unrestricted susceptible population*) showed 95% efficacy (95% CI 87–99%) regarding precancerous genital lesions, vulvar or vaginal, or condylomas and 98% efficacy (95% CI 92–100%) for CIN1+ or *in situ *adenocarcinoma caused by vaccine HPV types. The *intention-to-treat *analysis showed a 73% efficacy (95% CI, 58–83%) in preventing external anogenital or vaginal lesions of any degree and a 55% efficacy (95% CI, 40–66%) in preventing incident and prevalent cervical lesions of any grade associated with vaccine HPV genotypes, at 3 years follow up. In both FUTURE I and II studies, vaccination did not appear to significantly alter the course of infection or lesions due to HPV which were already present before the first dose of vaccine.

- *Per-protocol *efficacy analysis of the results achieved by the combination of three randomized clinical trials of vaccination (n = 7,811) versus placebo (n = 7,785) showed that vaccination, after an average follow-up period of 3 years, has an efficacy of 100% (95% CI 72–100%) in preventing vulvar and vaginal lesions grade 2 and 3 associated with HPV-16 and HPV-18. *Modified intention-to-treat *analysis demonstrated an efficacy of 97% (95% CI 79–100%) and *intention-to-treat analysis*, on a total of 18,174 randomised women, showed an efficacy of the vaccine at three years of 71% (95% CI, 37–88) in the prevention of incident/prevalent HPV-associated lesions [153].

- *Per-protocol *efficacy analysis of the results achieved by the combination of four randomized clinical trials of vaccination with the quadrivalent vaccine (n = 9,087) and its HPV-16 L1 component (n = 1,204) *vs*. placebo (n = 10,292) showed that, after a follow-up of 3 years, in HPV-16 and HPV-18 negative women, vaccine efficacy was 99% (95% CI 93–100) in preventing development of CIN2/3, *in situ *adenocarcinoma or invasive cervical cancer associated with HPV-16 and HPV-18. Efficacy was 98%, (95% CI 93–100) in the population of *modified intention-to-treat analysis*. The *modified intention-to-treat analysis *demonstrated an impact of the vaccine of 44% (95% CI, 31–55) in reducing the incidence of cervical lesions associated with HPV-16 and HPV-18, in the case of HPV type vaccine diseases already prevalent/incident in the population [157].

#### Efficacy of the AS04 adjuvanted bivalent VLP vaccine

Efficacy the AS04 adjuvanted bivalent VLP vaccine has been assessed in phase II-III studies. A pilot randomized phase II study on safety, immunogenicity, and efficacy of the vaccine for prevention of incident and persistent HPV-16 and HPV-18 infection enrolled and randomized 1,113 women aged 15–25 years to receive either the vaccine or placebo [144]. Study participants were initially seronegative for HPV-16 and HPV-18 by ELISA and negative for HPV-16 and HPV-18 DNA by PCR and had negative results at cervical cytology. In the *per protocol *analysis, vaccine efficacy was 91.6% (95% CI, 64.5%–98.0%) against incident infection and 100% against persistent infection (95% CI, 47%–100%) with HPV-16/18 at 18 months post-vaccination. In the *intention-to-treat *analyses, vaccine efficacy was 95.1% (95% CI, 63.5%–99.3%) against persistent cervical infection with HPV-16/18 and 92.9% (95% CI, 70%–98.3%) against cytological abnormalities associated with HPV-16/18 infection. In a subsequent report from the same study, after a follow-up of 4–5 years, *per protocol *analysis on the results obtained from a group of 732 women (367 in the vaccination group and 365 in the placebo group) who received all three doses of vaccine, demonstrated vaccine efficacy against incident infection was 96.9% (95% CI, 81.3%–99.9%) and 100% (33.6%–100%) against persistent infection for 12 months [145]. Efficacy against CIN lesions associated with HPV16/18 infection was 100% (95% CI, 42.4%–100%), since 8 cases of CIN1/2 occurred in the placebo group and none in the vaccination group. Interim results at a mean follow-up of 14.8 months (SD 4.9) from a large, international phase III study have been published [149]. In this trial, 18525 women aged 15–25 years from North American, South American, European, Asian, and Australian countries were randomly assigned to receive either HPV16/18 vaccine (n = 9,258) or hepatitis A vaccine (n = 9,267) at 0, 1, and 6 months. Two cases of CIN2+ associated with HPV16 or HPV18 DNA were seen in the HPV16/18 vaccine group; 21 were recorded in the control group. Based on these data, vaccine efficacy against CIN2+ containing HPV16/18 DNA was estimated to be 90.4% (97.9% CI, 53.4–99.3), but, a post hoc analysis demonstrated that the HPV16 or HPV18 DNA in the 2 CIN2+ cases in the vaccination group and in 1 case of the control group were already present at study entry. Efficacy against 12-month persistent HPV-16/18 infection was 75.9% (97.9% CI, 47.7–90.2).

#### Efficacy of HPV VLP vaccination in HPV-positive women

In women positive for HPV DNA, HPV VLP vaccination does not accelerate clearance of the virus nor prevents incident CIN2+ caused by HPV vaccine types. This finding has been demonstrated in a randomized phase III trial in 2189 women aged 18–25 years, who received the AS04 adjuvanted bivalent VLP vaccine [158] and in two randomized trials of the quadrivalent HPV vaccine enrolling 17,622 women without consideration of HPV status [159].

#### Cross-protection against oncogenic HPVs

Two clinical trials have evaluated the efficacy of Cervarix™ on other oncogenic types [144-146]. They demonstrated vaccination led to sustained cross-protection against incident infection with HPV-45 and -31 for the entire follow-up period up to 5.5 years and cross-protection against six-month persistent infection due to HPV-45, -31 and -52 [160]. Furthermore, broad protection was observed against 12-month persistent infections (defined as detection of the same HPV type in all available cytology samples collected over any 12-month period) with 12 combined oncogenic HPV types, not including HPV-16 and -18 (vaccine efficacy 27.1%; 97.9% CI: 0.5–46.8) [160]. The impact of Gardasil^® ^HPV quadrivalent vaccine on the rates of infection and disease associated with 10 HPV types not included in the vaccine was estimated in a population of women that was, pre-vaccination, seronegative and PCR-negative to all HPV types for which testing was available and had a normal Pap test [161]. Within approximately 3 years of follow-up, combined efficacy for CIN2/3 or adenocarcinoma in situ caused by all 10 non-vaccine HPV types was 38% (95% CI:6–60). The final end-of study data with approximately 3.6 years follow-up showed similar high vaccine efficacy [161].

### Side effects of prophylactic HPV VLP vaccines

Data on safety of the quadrivalent HPV VLP vaccine reported a higher rate of side effects in the vaccination group than in the placebo group. Side effects included local reaction at the site of injection (erythema, pain, edema) (altogether, 86.8% in the vaccine group vs. 77.4% in the placebo group, respectively) and systemic side effects, such as fever (13.5% vs. 10.2%, respectively) [149]. Also data on safety of the bivalent vaccine reported a higher rate of injection site symptoms and systemic side effects (fatigue, headache, myalgia) in the vaccine group than in the control group [146]. No increased incidence of spontaneous abortion, late fetal deaths, or congenital anomalies was observed in pregnancies occurring in women receiving either type of vaccines as compared with controls [146,148].

#### Recommendations on the use of prophylactic HPV vaccines

• HPV vaccination with the quadrivalent vaccine is safe and efficacious for the prevention of incident high-grade precancerous lesions, *in situ *adenocarcinoma, invasive cervical cancer, high-grade vulvar and vaginal lesions, and genital warts correlated with HPV-16, HPV -18, HPV -6, and HPV -11 (I).

• HPV vaccination with the AS04 adjuvanted bivalent vaccine is safe and efficacious for the prevention of incident and persistent HPV-16 e HPV-18 infection and CIN1+ correlated with HPV vaccine types (I)

• Long-term efficacy (> 5 years) of HPV vaccination is currently under investigation (research need).

• Efficacy of HPV vaccination has been demonstrated in women aged 15–26 years, who had not been previously exposed to HPV vaccine types (I); results on efficacy in women older than 26 years are not available yet (research need).

• Results on immunogenicity and safety (but not efficacy) justify vaccination in girls aged 9–14 years (II).

• Efficacy of HPV vaccination in males is currently under investigation (research need).

• HPV vaccination in males is not considered to be cost-effective (III).

• HPV vaccination should be done before exposure to genital HPV, since it lacks efficacy in women who have already been infected (IA), and it does not enhance virus clearance (IA).

• Screening for cervical cancer should be recommended both in vaccinated and non-vaccinated women according to current guidelines (II). Screening protocols could be modified only after appropriate clinical trials (research need).

• Further research is needed to assess the duration of vaccine protection, variations in the prevalence of HPV types in vaccinated women and in the general population, and surveillance of side-effects of vaccination (research need).

## Summary of recommendations and guidelines

### When should HPV testing be used in cervical-cancer screening programs?

• in ASCUS triage (IA).

• in the follow-up after treatment of CIN2/3 (IA).

• as a screening test for cervical cancer, as an alternative to cytology, in women older than 30 years, with cytology or repeat HPV testing as triage tests (IA).

Efficacy in cervical cancer screening has been proven for both PCR-based and signal amplification-based HPV tests (I).

### What is the role of new HPV tests in the diagnosis of HPV infection?

• Evaluation of HPV persistence may be useful to identify patients at risk for cervical cancer, who should be referred for colposcopy (A).

• HPV typing and subtyping can be used for epidemiological evaluation of HPV distribution and diffusion (B), for identification of persistent infection (B), for identification of HPV-16 and HPV-18, which are associated with a higher risk of precancerous lesions (B) or invasive cervical cancer (B), and for monitoring the efficacy of vaccination (A). But, there is insufficient evidence to define the best protocol to manage women with positive tests (I).

• Detection of high-risk HPV E6/E7 mRNA may be useful to identify women at risk for cervical cancer and with worse prognosis (IIB). Further research is needed to define the best protocol to manage women with a positive test and to evaluate test efficacy in primary screening programs.

• Measurement of HPV-16 DNA load in women with normal cytology may be useful to identify women at risk for cervical cancer (IIB). But, there is insufficient evidence to define the best protocol to manage women with positive tests (I).

• In epidemiological surveys, HPV serological testing and measurement of anti-HPV cellular immune response could be useful to monitor the duration of vaccine protection (B).

### Who should be vaccinated and how should vaccine efficacy be evaluated?

• Quadrivalent HPV VLP vaccine is safe and effective in the prevention of cervical and vulvar precancerous lesions, *in situ *adenocarcinoma, invasive cervical cancer and genital warts associated with vaccine HPV types in women aged 15–26 yrs (I).

• HPV vaccination with the AS04 adjuvanted bivalent vaccine is safe and efficacious for the prevention of incident and persistent HPV-16 e HPV-18 infection and CIN1+ correlated with HPV vaccine types in women aged 15–26 yrs (I)

• HPV vaccine should be administered before genital HPV exposure, since the benefit of vaccination declines in women already positive for HPV DNA (IA).

• In epidemiological surveys, HPV serological testing and HPV genotyping could be useful to monitor vaccine efficacy (research need).

• Cervical cancer screening is recommended both in vaccinated and non-vaccinated women, in agreement with current guidelines for cervical cancer prevention (III).

• Further research is needed to assess the duration of vaccine protection, variations in the prevalence of HPV types in vaccinated women and in the general population, and surveillance on side-effects of vaccination (research need).

## Competing interests

The authors declare that they have no competing interests.

## Authors' contributions

LB drafted the manuscript. CG, FMB, and GP contributed to the preparation of guidelines. All authors read and approved the final manuscript.
